# Machine Learning Techniques in Concrete Mix Design

**DOI:** 10.3390/ma12081256

**Published:** 2019-04-17

**Authors:** Patryk Ziolkowski, Maciej Niedostatkiewicz

**Affiliations:** Faculty of Civil and Environmental Engineering, Gdansk University of Technology, Gabriela Narutowicza 11/12, 80-233 Gdansk, Poland; mniedost@pg.edu.pl

**Keywords:** concrete, concrete mix design, concrete strength prediction, data mining, machine learning

## Abstract

Concrete mix design is a complex and multistage process in which we try to find the best composition of ingredients to create good performing concrete. In contemporary literature, as well as in state-of-the-art corporate practice, there are some methods of concrete mix design, from which the most popular are methods derived from The Three Equation Method. One of the most important features of concrete is compressive strength, which determines the concrete class. Predictable compressive strength of concrete is essential for concrete structure utilisation and is the main feature of its safety and durability. Recently, machine learning is gaining significant attention and future predictions for this technology are even more promising. Data mining on large sets of data attracts attention since machine learning algorithms have achieved a level in which they can recognise patterns which are difficult to recognise by human cognitive skills. In our paper, we would like to utilise state-of-the-art achievements in machine learning techniques for concrete mix design. In our research, we prepared an extensive database of concrete recipes with the according destructive laboratory tests, which we used to feed the selected optimal architecture of an artificial neural network. We have translated the architecture of the artificial neural network into a mathematical equation that can be used in practical applications.

## 1. Introduction

Concrete mix design is an essential and abstruse topic, which requires extensive knowledge of many expert issues. Obtaining concrete with appropriate strength, and other utility parameters, allows for the reliable use of the structure. The process of concrete hardening and hydration are irreversible. Therefore, any errors in the design of the concrete mix are incredibly costly for the investor, both at the construction stage and in the subsequent exploitation of the structure due to reduced durability. By definition, concrete mix is a mixture of cement, water, and coarse and fine aggregate, mostly enriched by additives and admixtures to improve some parameters, such as concrete strength, density, durability, or workability. The final product is in which the concrete mix is transformed into concrete. The concrete hardening is started by the cement hydration process, which is an exothermic chemical reaction between cement and water. Hydrated cement forms a tobermorite gel, hydroxide, and some secondary compounds that help with bonding between the fine and coarse aggregate. In the course of the hydration process, the hydration products gradually deposit on the original cement grains and fill the space occupied by water. The hydration process stops when there is no unreacted cement or the water molecules are retracted. The hardening of concrete continues further and ends around the twenty-eighth day, when the concrete reaches full compressive strength [1,2,3]. The necessary amount of water for full hydration of cement varies from 20% to 25% of its mass, without taking into account the water trapped in the pores [4,5]. According to Power’s model, the water required to hydrate cement entirely is 42% by weight [6,7]. The issue of concrete mix design boils down to selecting the correct proportions of cement, fine and coarse aggregate, and water to produce concrete that has the specified properties. Progress in the design of concrete mixes is at a moderate level. The most popular method used to estimate the amount of main ingredients needed, in a little changed form, has been used for decades and consists of estimating the strength of concrete mortar for bending [8,9,10]. These methods have many disadvantages and are labour-intensive to use. We want to introduce a way to design a concrete mix based on a mathematical equation developed by the machine learning algorithm. In the following paper, we describe the adopted neural network architecture, which further will be feed by a large dataset of concrete mix recipes. Finally, we present a mathematical formula that allows estimating the compressive strength of concrete. The developed formula will evaluate the compressive strength of concrete based on four input variables, the amount of cement, water, fine, and coarse aggregate. The presented equation does not reflect the behaviour of the concrete perfectly and has boundary conditions. However, it is a step on the way to the introduction of machine learning techniques for concrete mix design. In its present form, it can be a tool for a rough estimation of the concrete class. In our further endeavours we would like to emphasise concrete mix design in terms of durability and service life estimation, then the influence of concrete admixtures, such as superplasticizers, would be essential [11,12,13,14,15,16].

## 2. The Contemporary Concrete Mix Design and Machine Learning Techniques

### 2.1. Concrete Mix Design in European Corporate Practice

The primary goal of concrete mix design is to estimate the proper quantitative composition and proportion of concrete mixture components. We should use a composition which allows us to achieve the best possible concrete performance. Concrete performance is characterised by several features, from which the most significant are compressive strength and durability. Both concrete strength and durability should play an essential role in the concrete mix design. The issue of durability is essential in the case of an aggressive environment [17,18,19,20,21,22]. Based on industry experience, we found out that, in European corporate engineering practice, there are a few most used methods for designing a concrete mix. These methods are the Bukowski method, the Eyman and Klaus method, and the Paszkowski method. All the solutions mentioned above are derived from the so-called “Three Equations Method”, or Bolomey Method, which is a mixed experimental-analytical procedure [10,23,24]. It means that collected laboratory data should confirm that mathematical approach. We calculate a volume of required components by analytical measures and validate the results by destructive laboratory testing. In this method, we use a fundamental equation of strength, consistency, and tightness to determine the three searched values, as follows, the amount of aggregate, cement, and water, expressed in kilograms per cubic meter.

The first equation is the compressive strength equation or Bolomey formula (Equation (1)), which expresses the experimentally determined dependence of the compressive strength of hardened concrete on the grade of cement used, the type of aggregate used, and the water-cement ratio characterising the cement paste [23,24]. In this method, the concrete grade is assumed as input data.

(1) $$f_{cm}=A_{1,2}\left(\frac{C}{W}\pm 0,5\right)\;[\mathrm{MPa}],$$

where, $f_{cm}$ is a medium compressive strength of concrete, expressed in kilograms. The value $A_{1,2}$ means coefficients, depending on the grade of cement and the type of aggregate; C is an amount of cement in 1 m^3^ of concrete, expressed in kilograms; and W corresponds to the amount of water in 1 m^3^ of concrete, expressed in kilograms. A second consistency Equation (2), is included in the water demand formula necessary to make a concrete mix with the required consistency.

(2) $$W=C\cdot w_{c}+K\cdot w_{k}\;[{\mathrm{dm}}^{3}],$$

where W is the amount of water in 1 m^3^ of concrete, expressed in kilograms; C corresponds to the amount of cement in 1 m^3^ of concrete, expressed in kilograms; K means the amount of aggregate in 1 m^3^ of concrete, expressed in kilograms; $w_{c}$ is the cement water demand index in dm^3^ per kilogram; and $w_{k}$ is the aggregate water demand index in dm^3^ per kilogram. The water-tightness of concrete Equation (3) is included in the simple volume formula, which indicates that a watertight concrete mix is obtained if the sum of the volume of the individual components is equal to the volume of the concrete mix.

(3) $$\frac{C}{\rho_{c}}+\frac{K}{\rho_{k}}+W=1000\;[{\mathrm{dm}}^{3}],$$

where W is the amount of water in 1 m^3^ of concrete, expressed in kilograms, C corresponds to the amount of cement in 1 m^3^ of concrete, expressed in kilograms, K means the amount of aggregate in 1 m^3^ of concrete, expressed in kilograms;$\;\rho_{c}$ is the cement density in kilograms per dm^3^; and $\rho_{k}$ is the aggregate density in kilograms per dm^3^.

The system of equations presented above, with three unknowns variables, allows for calculating the sought amounts of cement (C), aggregate (K), and water (*W*) in one cubic meter of concrete mix. The system is valid, assuming that there are no air bubbles in the concrete. Another method used in the construction industry is “the double coating method” [25]. The methods above are ones that are used to determine the quantitative composition of the concrete mix. However, the actual process of creating a concrete mix is much broader, including the following steps: The first step is to determine the data needed to design the mix, such as the purpose of the concrete use, the compressive strength of the concrete, and the consistency of the concrete mix. Next, the qualitative characteristics of the components should be determined, namely the type and class of cement and the type and granularity of the aggregates. Subsequent steps include an examination of the properties of the adopted ingredients; a check of their compliance with the standard requirements; determining the characteristics of the components that will be needed to determine the composition of the concrete mix; and a projection of the aggregate pile. The successive step is the actual adoption of the design method and a calculation per unit of volume. The final stage is to make a trial sample and examine both the concrete mix and the hardened concrete with design assumptions [26].

### 2.2. Machine Learning Techniques

#### 2.2.1. The Overall Concept of Machine Learning

Machine learning is an area of knowledge which is developing dynamically in recent times. This technology is a part science dealing with artificial intelligence and refers to scientific fields such as computer science, robotics, and statistics [27]. In practice, machine learning aims to use various state-of-the-art achievements in computer science to build upon a system that will be able to learn from data sets and, thus, seek patterns and relationships between variables and groups of variables, which would be challenging to conduct with conventional methods. Learning, in this case, can be considered as the instantiation of the sophisticated algorithm. One of the most popular methods of machine learning is artificial neural networks (ANN).

#### 2.2.2. Artificial Neural Networks (ANN)

ANN are clusters of neurons, which are also its basic unit. We can consider an artificial neuron as a specific signal converter. The behaviour of artificial neurons, in a sense, imitates the behaviour of neurons in the human brain [28]. A primary example of ANN consist of three layers, called as follows:

- The input layer;
- The hidden layer;
- The output layer.

The input layer consists of input variables and combines them with neurons from the hidden layer. On the contrary, the output layer contains the target data to be obtained by the hidden layer [27]. Therefore, the whole process of learning happens in the hidden layer, where connections between neurons are sought. Vast numbers of neurons can build a complex model, which would be unattainable with simple architecture and so unobvious that it would be difficult to create a purely empirical formula. An essential thing that neural networks do is a search for patterns, which is why examples best teach neural networks. To teach a neural network how to solve a given problem, one must enter the input data into it using the first layer and put data in the output layer as a given target to which the network is to strive. Moreover, the input data can be adjusted by assigning weight to them, which can potentially represent the importance of a given variable. The weight control mechanism is also part of the neural network and is called the “learning rule”. One artificial neuron has miserable problem-solving capabilities. Many neurons can be combined into more hidden layers, where layers pass the results to one another, looking to reach the target value [29,30].

### 2.3. Use of Machine Learning in Concrete Compressive Strength Prediction

Designing a concrete mix consists of selecting components and their amount to achieve specific parameters of the concrete. One of the most significant parameters for concrete performance is the compressive strength of concrete, which defines the class of concrete. Other important parameters that contribute to good concrete performance are durability and even the manufacturing process itself. Poor durability may contribute to lowering the service quality of building in time. With a wrong manufacturing process, for example, poor concrete care can cause excessive cracks and reduce concrete tightness [31].

The issue of machine learning applications, more precisely ANN, to predict the strength of concrete is present in the scientific discourse and is continuously evolving, making this topic very progressive.

The topic was first discussed in 1998 by Yeh et al. [32], which used linear regression and ANN to try to predict the strength of high-performance concrete using seven input variables. In the research, Yeh et al. used an extensive database, but in our opinion, they did not take into account the specificity of concrete and used samples in their database that were still in the maturing phase, even three days old, which, in our opinion, could seriously misrepresent the results.

Subsequently, the topic was taken up by Seung-Chang Lee [33], which used a modular network structure consisting of five ANN. In the presented solution, the author used the weighting technique of input neurons to improve the accuracy of predictions. To estimate the number of input neurons, he used the parameter condensation technique. The author concludes that the methods he uses, namely condensation and weighting techniques, are efficient in looking for the optimal performance network.

Another interesting approach in this matter is to use a neural-expert system, which was suggested further by Gupta et al. [34] to predict the compressive strength of high-performance concrete. The neural expert system architecture, in theory, allows for constructing the database automatically by learning from example inferences. In general, this architecture assumes the use of a multi-layered neural network, which is consequently trained with generalised backpropagation for interval training patterns. However, this may allow for the learning of patterns with irrelevant inputs and outputs. What is more, in the study by Gupta et al. [34], the input variables have very different input metrics and instead of the amounts of concrete mix components, the input variables refer to such parameters as curing time. In our opinion, the selected input parameters have no unambiguous effect on the strength of concrete and can imply false results. The topic of neural-expert systems was also undertaken by Dac-Khuong Bui et al. [35], which focused entirely on the practical application of the mentioned expert approach.

Fangming Deng et al. [36] practised deep learning architecture to predict the compressive strength of concrete. In this study their used recycled concrete with five input variables as follows, water-cement ratio, recycled coarse aggregate replacement ratio, recycled fine aggregate replacement ratio, and fly ash replacement. They used so-called deep features that refer to ratio rather than the individual amount of concrete mix components. We used a similar approach in our study by introducing feature scaling. To find out the proper prediction model they used a Softmax regression. In the results section of their paper, they state that the deep learning architecture they applied gives a higher efficiency, generalisation ability, and precision, in comparison with standard ANN. However, they do not present sufficient proofs to support their statements. Convolution networks are computationally expensive. This seems to be confirmed by a significantly lower number of samples (74 exactly) than in our study (741 records). However, such a small dataset might result in underfitting, which means that the model does not fit the data well enough to such an extent that it reduces the efficiency of the model. Moreover, Hosein Naderpour et al. [37] shows a comparable degree of precision between ANN and Deep Neural Networks (DNN).

## 3. Materials and Methods

### 3.1. Essentials

**Figure 1:**
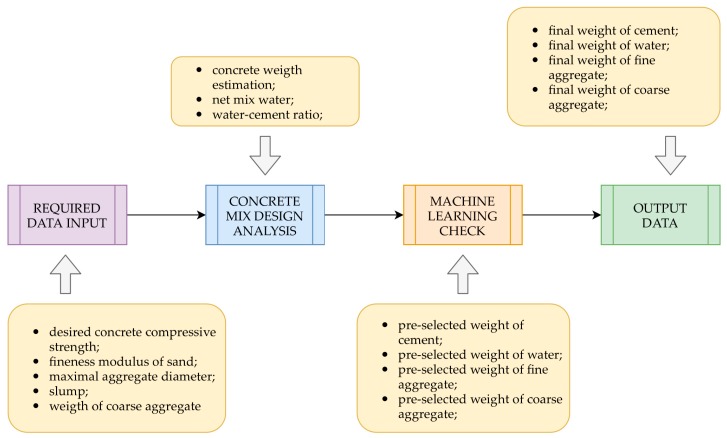
Block diagram of the practical application of machine learning in the concrete mix design.

In our study, we want to implement machine learning for concrete mix design. Based on a large number of tested concrete mix recipes, we would like to build an ANN which will be able to estimate the compressive strength of the concrete mix. The ANN estimates the strength of the concrete based on the amount of the four main components of a concrete mix, more precisely cement, fine and coarse aggregate, and water. We translated the constructed ANN into the source code and simplified to one equation, defining the twenty-eight-day strength of concrete as a function of the four parameters. The equation can be used for concrete compressive strength estimation and can serve as a tool for a concrete mix recipe check. The practical application of this method in the concrete mix design process, required to adopt the approach, is presented in Figure 1.

It seems reasonable to set a boundary condition for this method. However, the ANN was trained on a limited number of samples so it may be difficult to predict how it will behave for amounts of material higher than in the considered ranges. It is essential to strictly control the water-cement ratio since the proper proportion is necessary for the full hydration of the cement. We have not analysed the influence of plasticisers.

### 3.2. The Database of Concrete Mix Recipes

In our research, we intend to teach the neural network the relationships between the number of individual components in a concrete mix and the compressive strength of concrete with a large number of examples. Thanks to this, the potential user of our solution will be able to design the right composition of ingredients and try to predict the compressive strength of concrete. To handle that task, we need a wide-ranging database containing a variety of concrete recipes with according data of their destructive laboratory tests. We prepared the database, which has many records from numerous sources, including literature, companies, institutions, and laboratories. The concrete mix recipes that we used for the analysis were designed for concrete structures of different dimensions, functions, and destinations. Therefore, there may be some differences between them, the sources of which we will not be able to predict. What is more, many of the recipes we have, besides the essential ingredients, have additives that have different functions. The most popular concrete additives are binding retardants, plasticisers, and workability boosters. The samples tested are standardised concrete cylinders with a diameter of 15 cm. Samples that were not cylindrical were converted into cylindrical ones according to valid norms [38]. The size of the aggregate in the dataset did not exceed 20 mm. The samples were made from normal Portland cement. We have carried out extensive consultations with experts and have adopted four components that have a significant impact on the compressive strength of concrete. The adopted input parameters are presented in Table 1.

We divided the parameters from Table 1 into two groups, the inputs and target, which characterise input and output variables, respectively. After initiating the cement hydration process, concrete strength grows, progressively over time, to full strength. In our deliberations, we adopted a general assumption that concrete achieves its designed compressive strength in twenty-eight days. Prior to the twenty-eighth day, the concrete has a partial strength, but it cannot be considered full strength. We assumed in our research that the concrete reached its full strength because a mixture is designed for such strength. We removed all records for concrete of lower ages from the base. Many factors have an indirect effect on the obtained concrete strength, which has not been included in the analysis, such as the curing process. We assumed that quality control was sufficient to produce full strength concrete. The minimum, maximum, and average values for every input variable are presented in Table 2.

### 3.3. Results and Discussion

**Figure 2:**
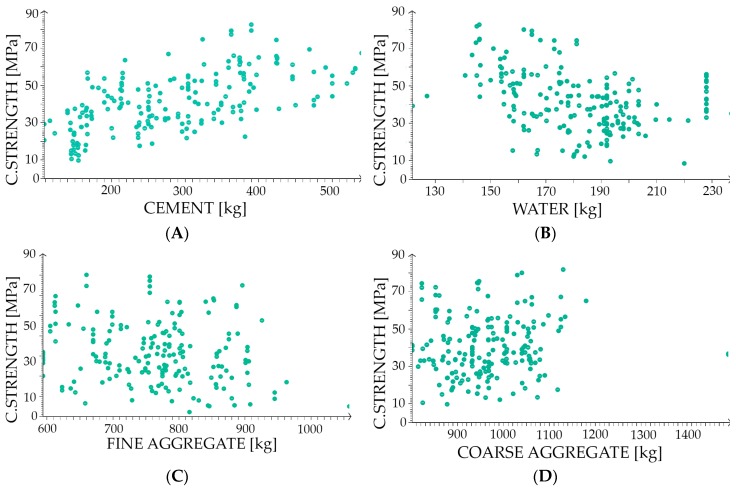
The scatter plots target versus input variables. The vertical axis is the full compressive strength of concrete expressed in megapascals. The horizontal axis is the amount of material in kilograms for cement and aggregate as well as in litres for water. (**A**) Cement; (**B**) water; (**C**) Fine aggregate (sand 0–2 mm); and (**D**) coarse aggregate (aggregate above 2 mm). The legend is in the left bottom corner.

To carry out the simulation, we divided our set into three subsets, as follows: The training dataset, the selection dataset, and the testing dataset. The training dataset is used to create a neural network, the selection dataset is used to adjust parameters of the neural network, and the testing dataset is used to evaluate the efficiency of the network. The database has 741 records, but we had to exclude 79 records (10.7%) from the analysis as univariate outliners. The training dataset has 395 records (53.3%), the selection dataset has 133 records (17.9%), and the testing dataset has 134 records (18.1%). The scatter plots of a target variable versus the input variables are presented in Figure 2.

**Figure 3:**
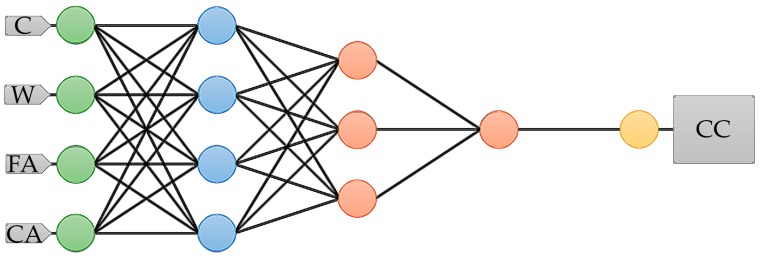
The initially used architecture of the ANN. The figure shows the network architecture, which includes the following parts, principal components (blue), perceptron neurons (red), and, because we use feature scaling, there are scaling and unscaling layers. The scaling and unscaling neurons are green and yellow, respectively. Abbreviations: C, cement; W, water; FA, fine aggregate; CA, coarse aggregate; CC, full compressive strength of concrete.

Our neural network consists of four input variables, which refers to four principal components and generates one target output. The complexity of the model is expressed by the number of hidden layers, which in our case is three. The initial architecture that we prepared is shown in Figure 3, which consists of principal components (blue), perceptron neurons (red), and, because we used feature scaling, there are scaling and unscaling layers. The scaling and unscaling neurons are green and yellow, respectively. 

We want to point out that some input variables (cement, water, fine_aggregate, coarse_aggregate) correspond with some input neurons and target variable (cs_28) is associated with the output neuron. To obtain a proper training rate, we used the Broyden-Fletcher-Goldfarb-Shanno algorithm [39,40,41,42,43,44]. Then, to designate the quasi-Newton training direction step, we utilised the Brent method [45,46,47,48]. For the analysis, we calculated the linear correlation and determined a correlation matrix.

**Figure 4:**
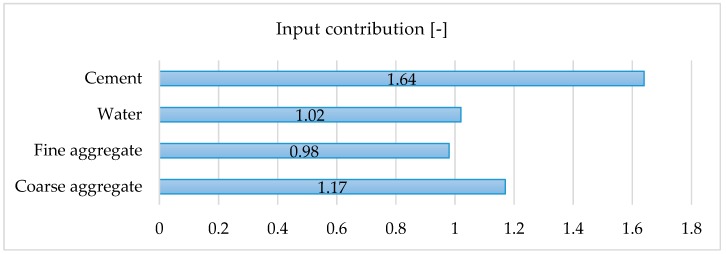
Input contribution.

We have assessed the impact of individual variables on the final result, which is presented in Figure 4. We eliminated training input selectively and inspected the output results. An input contribution value 1.0 or lower than one denotes that the variable has less contribution to the results. Successively, a value higher than 1.0 means a more significant contribution. Our analysis indicates that the biggest contribution to the results have cement, which is in line with our assumptions that the water-cement ratio has the most significant impact on concrete strength. Literature findings also confirm that the cement content and type have a high influence on the compressive strength [49]. There are also other issues, including curing conditions and added admixture impact, that influence the compressive strength and concrete durability, especially an environments with a high risk of carbonation [50]. The detailed nature and the shape of the aggregate influence the workability and durability of concrete. The shape and texture of the aggregate affect the properties of fresh concrete more than hardened concrete [51]. Additionally, the grading or size distribution of aggregate is an important characteristic because it determines the paste requirement for workable concrete [52]. However, in our procedure, we did not make an exact distinction between the nature and shape of the aggregate. We only diversified the coarse and fine aggregates and sacrificed it for the sake of having larger data sample pools in these two categories. We also have not analysed the impact of environmental aggression and admixtures.

**Figure 5:**
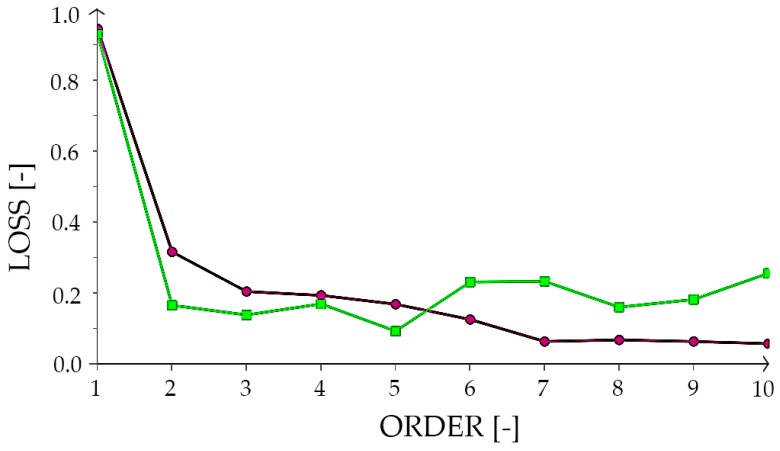
Incremental order algorithm performance. The chart presents a loss history, where the purple line is the training loss and the green one is the selection loss. The vertical axis is a loss and the horizontal axis is an order.

We performed input selection by the growing inputs algorithm [53,54,55,56]. We found the optimal number of neurons by the order selection algorithm [57,58]. We carried out the output selection by the incremental order algorithm [59,60,61]. The loss history for the subsets used is presented in Figure 5.

**Figure 6:**
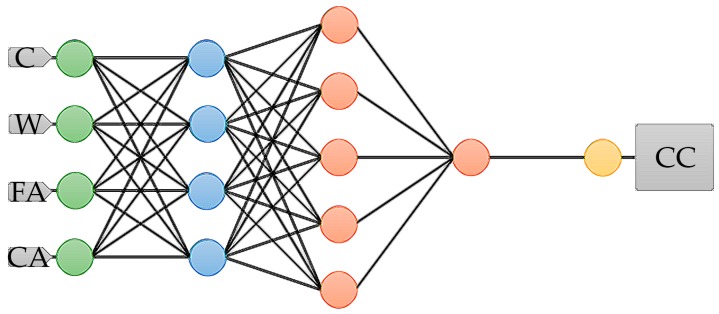
Finally used the architecture of ANN. The figure shows network architecture that includes following parts, principal components (blue), perceptron neurons (red), and, because we use feature scaling, there are scaling and unscaling layer. The scaling and unscaling neurons are green and yellow, respectively. Abbreviations: [C]—cement; [W]—water; [FA]—fine aggregate; [CA]—coarse aggregate; [CC]—full compressive strength of concrete.

In Figure 6 we present a final architecture of the ANN, which consists of principal components (blue), perceptron neurons (red), and, because we used feature scaling there are scaling and unscaling layers. The scaling and unscaling neurons are green and yellow, respectively. We used a deep architecture with features scaling. Therefore it contains scaling and unscaling layers. Our final model, which is the most optimal for performing the given task, has four inputs, one output, and three hidden layers.

In our study, we created an ANN which can be used for concrete mix design. The network targets the compressive strength of concrete with the four following input variables, cement, water, fine, and coarse aggregate. We can express our ANN by a mathematical Equation (4). The Equation (4) refers to the 28 day strength of concrete, which, as we mentioned, can be considered as full strength.

(4) $$f_{c}^{full\;cs}=0.5\cdot \left(f_{ax}^{full\;cs}+1.0\right)\cdot \left(62.62184+240.13674\right)-240.13674\;[\mathrm{MPa}],$$

Auxiliary mathematical formulas are presented in Equations (5)–(14). The variables C, W, FA, CA used in Equations (15)–(18) mean cement, water, fine aggregate, and coarse aggregate, respectively. Units are expressed in kilograms per cubic meter.

(5) $$f_{ax}^{full\;cs}=tanh\left(0.246033-0.959961\cdot ax_{11}+0.816467\cdot ax_{12}+0.526611\cdot ax_{13}\;+0.73407\cdot ax_{14}-0.270081\cdot ax_{15}\right)$$

(6) $$ax_{11}=tanh\left(0.15979-0.00384521\cdot ax_{21}+0.837402\cdot ax_{22}-0.148804\cdot ax_{23}\;-0.569336\cdot ax_{24}\right)$$

(7) $$ax_{12}=tanh\left(0.1297+0.820862\cdot ax_{21}+0.0808105\cdot ax_{22}+0.206116\cdot ax_{23}\;-0.601257\cdot ax_{24}\right)$$

(8) $$ax_{13}=tanh\left(0.262573-0.0964355\cdot ax_{21}+0.610413\cdot ax_{22}-0.380981\cdot ax_{23}\;+0.150024\cdot ax_{24}\right)$$

(9) $$ax_{14}=tanh\left(0.0534668+0.3526\cdot ax_{21}+0.929932\cdot ax_{22}-0.734924\cdot ax_{23}\;-0.415405\cdot ax_{24}\right)$$

(10) $$ax_{15}=tanh\left(-0.927551-0.993103\cdot ax_{21}-0.202698\cdot ax_{22}-0.719788\cdot ax_{23}\;-0.637817\cdot ax_{24}\right)$$

(11) $$ax_{21}=0.984539\cdot C_{ax}-0.144211\cdot W_{ax}-0.0968967\cdot FA_{ax}+0.0222878\cdot CA_{ax}$$

(12) $$ax_{22}=-0.117904\cdot C_{ax}-0.946362\cdot W_{ax}+0.249228\cdot FA_{ax}+0.168472\cdot CA_{ax}$$

(13) $$ax_{23}=0.0747692\cdot C_{ax}-0.00246874\cdot W_{ax}+0.576173\cdot FA_{ax}-0.813897\cdot CA_{ax}$$

(14) $$ax_{24}=0.105786\cdot C_{ax}+0.28913\cdot W_{ax}+0.772348\cdot FA_{ax}+0.555601\cdot CA_{ax}$$

(15) $$C_{ax}=\frac{2\cdot \left(C-86\right)}{454}-1$$

(16) $$W_{ax}=\frac{2\cdot \left(W-121.8\right)}{125.5}-1$$

(17) $$FA_{ax}=\frac{2\cdot \left(FA-372\right)}{957}-1$$

(18) $$CA_{ax}=\frac{2\cdot \left(CA-597\right)}{893}-1$$

We simplified the mathematical formula translated from the ANN source code and presented it in the form of Equation (19), $f_{c}^{\mathrm{full}\;\mathrm{cs}}$ with four variables C, W, FA, CA, which represent cement, water, fine aggregate, and coarse aggregate, respectively.

(19) $$\begin{array}{l} f_{c}^{full\;cs}=151.37929\cdot tanh(0.24603-0.95996\cdot tanh(-0.00077\cdot C-0.01524\cdot W-\\ 0.00066\cdot FA-0.00012\cdot CA+3.90060)+0.816467\cdot tanh(0.00331\cdot C-0.00588\cdot \\ W-0.00085\cdot FA-0.00105\cdot CA+1.99855)+0.526611\cdot tanh(-0.00079\cdot C-\\ 0.00828\cdot W+0.00012\cdot FA+0.00111\cdot CA+0.78030)+0.73407\cdot tanh(0.00061\cdot C-\\ 0.01672\cdot W-0.00114\cdot FA+0.00119\cdot CA+2.67645)-0.270081\cdot tanh(-0.00474\cdot \\ C+0.00243\cdot W-0.00180\cdot FA+0.00039\cdot CA+1.22858)\;)-88.757459\;[\mathrm{MPa}], \end{array}$$

**Figure 7:**
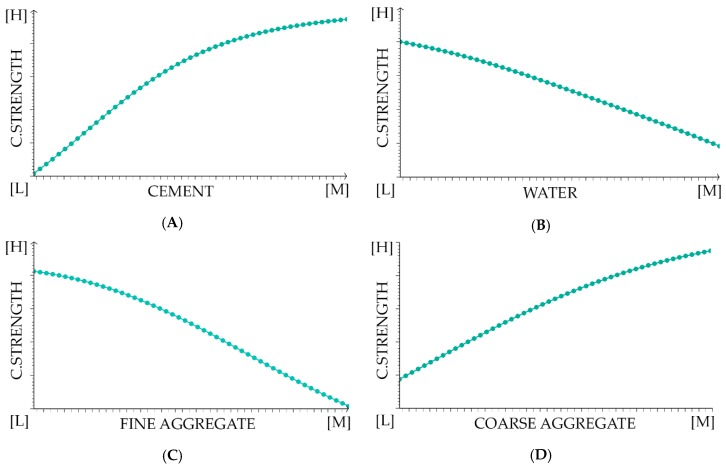
Output charts. The diagrams show variations in output variables for a single input variable, while the others are fixed. The vertical axis is the full compressive strength of concrete. The horizontal axis is the amount of material. (**A**) Cement; (**B**) water; (**C**) fine aggregate (sand 0–2 mm); (**D**) coarse aggregate (aggregate above 2 mm); [H], [M]—Higher/More; [L]—Lower/Less.

To illustrate how the equation works we presented the charts of the output variable and the single input variable, while the other input variable is fixed. The charts are shown in Figure 7. It should be noted that, as presented in Figure 7, the output charts do not correspond to the combined correlation of the variables, but only show a trend of a given variable concerning the target variable. It also should be noted that the parameters give a different contribution to the final results, as we have shown in Figure 4.

**Figure 8:**
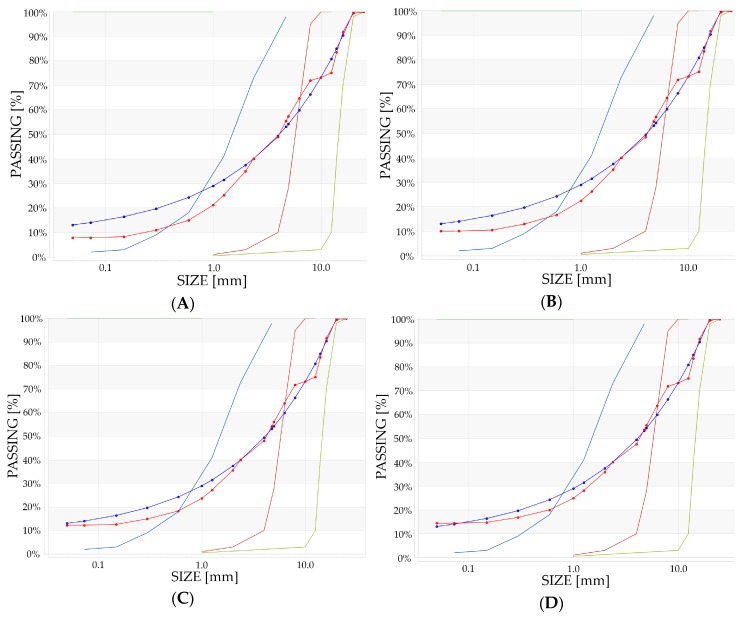

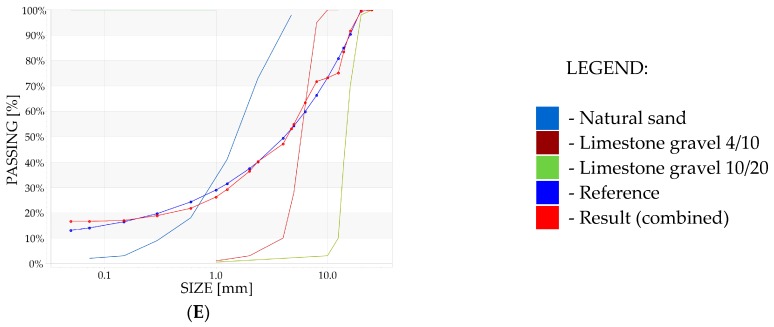
Gradings and fitting curves for designed concrete recipes: (**A**) 10 MPa; (**B**) 20 MPa; (**C**) 30 MPa; (**D**) 40 MPa; (**E**) 50 MPa; Legend in the left bottom corner.

**Figure 9:**
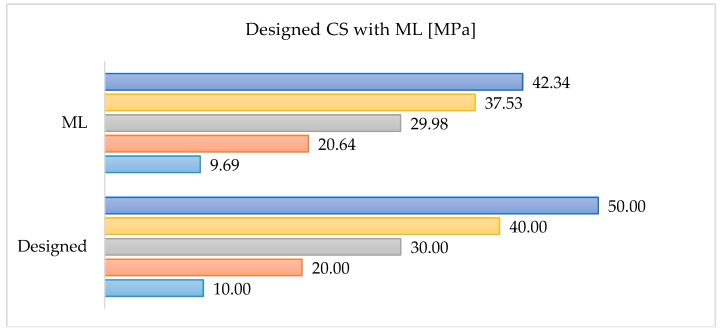
Comparison between designed Compressive Strength (CS) and calculated from Machine Learning (ML) formula.

We compared the presented Formula (19) with a standard concrete mix design approach, based on the Bolomey design method. The comparison was prepared for 1 m^3^ of concrete designed for the concrete slab, with direct pouring, plastic slump, no special desired finishing, no special ambient conditions when casting, and negligible environmental aggression. To design a concrete mix, we used the following materials: Portland cement; network water; natural sand; limestone gravel 4/10 mm; and limestone gravel 10/20 mm. The tested recipes are presented in Table 3. The gradings and fitting curves for the designed recipes are shown in Figure 8. The comparison is presented in Figure 9.

We observed a low resilience of the ANN formula for recipes of high strength (50 MPa and above) concrete. It may be due to the smaller number of recipes on which we trained the ANN for these ranges. This behaviour of the ANN may be a sign of underfitting [27,28]. We must point out that the presented method is only an introduction to the broader use of machine learning in the design of concrete mixes and does not exhaust this topic. In particular, it does not take into account some crucial issues, such as durability and the technological process.

## 4. Conclusions

Our study is focusing on the application of machine learning in concrete mix design and building a practical tool that could be used in engineering practice. We designed the optimal ANN architecture and fed it with an extensive database of concrete mix recipes for the study. Every concrete mix recipe record has a corresponding laboratory destructive test. While building a neural network, the goal was to predict the compressive strength of the concrete resulting from a specific composition of concrete mix ingredients, or more precisely, what ratio of ingredients should be selected to obtain concrete with an adequate compressive strength. Our database has 741 records. We excluded 79 (10.7%) concrete samples from the dataset, as univariate outliners. The specificity of machine learning requires us to divide the database into three subsets, which we split up as follows: The training subset has 395 records (53.3%), the selection subset has 133 records (17.9%), and the testing subset has 134 records (18.1%). The initially adopted ANN model has four input variables, four principal components, four hidden neurons, and one target output. The suitable training rate and the step for the quasi-Newton training direction were calculated by the Broyden–Fletcher–Goldfarb–Shanno algorithm and the Brent method, respectively. Our input contribution analysis discloses that the most significant impact on the obtained results is the amount of cement that accurately points out the significance of the water-cement ratio to reach a higher concrete compressive strength. The finally adopted ANN model has four input variables, four principal components, six hidden neurons, and one target output. The pivotal point in making the machine learning techniques more applicable was a transformation of the ANN into an actual mathematical equation, which can be used in engineering practice. The initial conversion of the ANN into the mathematical formula had fifteen equations and required fourteen auxiliary variables. We simplified the expression into one general equation for the calculation of twenty-eight-day compressive strength of concrete. The equation we developed can be used as a rapid tool for concrete mix design check. The method allows checking the composition of four main concrete mix ingredients, cement, water, fine, and coarse aggregate, for achieving the desired concrete class. However, we would like to denote that the presented mathematical expression does not adequately reflect all the relationships between the components and have certain boundary conditions. We want to further develop the presented method. In the next step, to make this method more reliable, we would like to analyse the effect of admixtures and concrete durability.

## Figures and Tables

**Table 1:** The parameters adopted in the dataset.

Parameter	Compressive Strength after 28 days	Cement	Water	Sand 0–2 mm	Aggregate above 2 mm
Codename	cs_28	cement	water	fine_aggregate	coarse_aggregate
Type	target	input	input	input	input
Description	The compressive strength of concrete 28 days after hydration. Considered as full strength.	The weight of cement added to the mixture	The weight of water added to the mixture	The weight of sand added to the mixture	The weight of aggregate, which have more than 2 mm, added to the mixture

**Table 2:** Ranges of input features of database input features.

Input Features	Minimum (kg/m^3^)	Maximum (kg/m^3^)	Average (kg/m^3^)
Cement	86.00	540.00	278.00
Water	121.80	247.00	182.42
Fine aggregate (sand 0–2 mm)	372.00	1329.00	768.55
Coarse aggregate (aggregate above 2 mm)	597.00	1490.00	969.08

**Table 3:** Tested concrete mix recipes.

Designed CC of Concrete Mix	Cement (kg/m^3^)	Water (L/m^3^)	Natural Sand (kg/m^3^)	Limestone Gravel 4/10 (kg/m^3^)	Limestone Gravel 10/20 (kg/m^3^)
10	190.42	61.89	1089.78	531.24	676.11
20	249.52	81.09	991.52	541.32	661.89
30	308.62	94.81	901.23	554.15	651.75
40	367.71	101.25	821.33	570.82	646.97
50	426.81	107.97	745.14	583.21	641.99

## References

[1] Marchon D., Flatt R.J. (2016). Mechanisms of cement hydration. Science and Technology of Concrete Admixtures.

[2] Scrivener K., Snellings R., Lothenbach B. (2018). A Practical Guide to Microstructural Analysis of Cementitious Materials.

[3] Kurdowski W. (2014). Cement and Concrete Chemistry.

[4] Young J.F., Mindess S., Darwin D. (2002). Concrete.

[5] Hover K.C. (2011). The influence of water on the performance of concrete. Constr. Build. Mater..

[6] Jensen O.M., Hansen P.F. (2001). Water-entrained cement-based materials: I. Principles and theoretical background. Cem. Concr. Res..

[7] Jensen O.M., Hansen P.F. (2002). Water-entrained cement-based materials: II. Experimental observations. Cem. Concr. Res..

[8] Jiménez Fernández C.G., Barra Bizinotto M., Valls del Barrio S., Aponte Hernández D.F., Vázquez Ramonich E. (2014). Durability of recycled aggregate concrete designed with the Equivalent Mortar Volume (EMV) method: Validation under the Spanish context and its adaptation to Bolomey methodology. Mater. Construcción.

[9] Abdelgader H.S. (1999). How to design concrete produced by a two-stage concreting method. Cem. Concr. Res..

[10] Abdelgader H.S., Suleiman R.E., El-Baden A.S., Fahema A.H., Angelescu N. Concrete Mix Proportioning using Three Equations Method (Laboratory Study). Proceedings of the UKIERI Concrete Congress Innovations in Concrete Construction.

[11] Cartuxo F., De Brito J., Evangelista L., Jiménez R.J., Ledesma F.E. (2016). Increased Durability of Concrete Made with Fine Recycled Concrete Aggregates Using Superplasticizers. Materials.

[12] Serralheiro M.I., de Brito J., Silva A. (2017). Methodology for service life prediction of architectural concrete facades. Constr. Build. Mater..

[13] Plank J., Sakai E., Miao C.W., Yu C., Hong J.X. (2015). Chemical admixtures—Chemistry, applications and their impact on concrete microstructure and durability. Cem. Concr. Res..

[14] Huang H., Qian C., Zhao F., Qu J., Guo J., Danzinger M. (2016). Improvement on microstructure of concrete by polycarboxylate superplasticizer (PCE) and its influence on durability of concrete. Constr. Build. Mater..

[15] Arredondo-Rea S.P., Corral-Higuera R., Gómez-Soberón J.M., Gámez-García D.C., Bernal-Camacho J.M., Rosas-Casarez C.A., Ungsson-Nieblas M.J. (2019). Durability parameters of reinforced recycled aggregate concrete: Case study. Appl. Sci..

[16] Andrade C., Gulikers J., Marie-Victoire E. (2018). Service Life and Durability of Reinforced Concrete Structures.

[17] Aïtcin P.C. (2003). The durability characteristics of high performance concrete: A review. Cem. Concr. Compos..

[18] Ahmad S. (2003). Reinforcement corrosion in concrete structures, its monitoring and service life prediction—A review. Cem. Concr. Compos..

[19] Schabowicz K., Gorzelańczyk T., Szymków M. (2019). Identification of the Degree of Degradation of Fibre-Cement Boards Exposed to Fire by Means of the Acoustic Emission Method and Artificial Neural Networks. Materials.

[20] Godycki-Ćwirko T., Nagrodzka-Godycka K., Wojdak R. (2016). Reinforced concrete thin-wall dome after eighty years of operation in a marine climate environment. Struct. Concr..

[21] Schabowicz K., Gorzelańczyk T., Szymków M. (2019). Identification of the degree of fibre-cement boards degradation under the influence of high temperature. Autom. Constr..

[22] Argiz C., Moragues A., Menéndez E. (2018). Use of ground coal bottom ash as cement constituent in concretes exposed to chloride environments. J. Clean. Prod..

[23] Rajamane N.P., Ambily P.S., Nataraja M.C., Das L. (2014). Discussion: Modified Bolomey equation for strength of lightweight concretes containing fly ash aggregates. Mag. Concr. Res..

[24] Zhang X., Deng S., Deng X., Qin Y. (2007). Experimental research on regression coefficients in recycled concrete Bolomey formula. J. Cent. South Univ. Technol..

[25] Abdelgader H.S., Saud A.F., Othman A.M., Fahema A.H., El-Baden A.S. (2014). Concrete mix design using the double-coating method. Betonw. Fert. Plant Precast Technol..

[26] Rumman R., Bose B., Emon M.A.B., Manzur T., Rahman M.M. An experimental study: Strength prediction model and statistical analysis of concrete mix design. Proceedings of the International Conference on Advances in Civil Engineering.

[27] Pereira F.C., Borysov S.S., Antoniou C., Dimitriou L., Pereira F. (2019). Machine Learning Fundamentals. Pereira Big Data and Transport Analytics.

[28] Shobha G., Rangaswamy S., Gudivada V.N., Rao C.R. (2018). Machine Learning. Computational Analysis and Understanding of Natural Languages: Principles, Methods and Applications.

[29] Yang Z.R., Yang Z., Araghinejad S. (2014). Artificial Neural Networks. Comprehensive Biomedical Physics.

[30] Silva I.N., Hernane Spatti D., Andrade Flauzino R., Liboni L.H.B., dos Reis Alves S.F. (2017). Artificial Neural Networks.

[31] Brown C.J. (2003). Design of Reinforced Concrete Structures.

[32] Yeh I.-C. (1998). Modeling of Strength of High-Performance Concrete Using Artificial Neural Networks. Cem. Concr. Res..

[33] Lee S. (2003). Prediction of concrete strength using artificial neural networks. Eng. Struct..

[34] Gupta R., Kewalramani M.A., Goel A. (2006). Prediction of Concrete Strength Using Neural-Expert System. J. Mater. Civ. Eng..

[35] Bui D.K., Nguyen T., Chou J.S., Nguyen-Xuan H., Ngo T.D. (2018). A modified firefly algorithm-artificial neural network expert system for predicting compressive and tensile strength of high-performance concrete. Constr. Build. Mater..

[36] Deng F., He Y., Zhou S., Yu Y., Cheng H., Wu X. (2018). Compressive strength prediction of recycled concrete based on deep learning. Constr. Build. Mater..

[37] Naderpour H., Rafiean A.H., Fakharian P. (2018). Compressive strength prediction of environmentally friendly concrete using artificial neural networks. J. Build. Eng..

[38] Toniolo G., di Prisco M. (2017). Reinforced Concrete Design to Eurocode 2.

[39] Abdi F., Shakeri F. (2017). A globally convergent BFGS method for pseudo-monotone variational inequality problems. Optim. Methods Softw..

[40] Andrei N. (2018). An adaptive scaled BFGS method for unconstrained optimization. Numer. Algorithms.

[41] Battiti R., Masulli F. (1990). BFGS optimization for faster and automated supervised learning. International Neural Network Conference.

[42] Berahas A.S., Nocedal J., Takác M. (2016). A multi-batch l-bfgs method for machine learning. Advances in Neural Information Processing Systems.

[43] Hagan M.T., Menhaj M.B. (1994). Training feedforward networks with the Marquardt algorithm. IEEE Trans. Neural Netw..

[44] Li D.-H., Fukushima M. (2001). A modified BFGS method and its global convergence in nonconvex minimization. J. Comput. Appl. Math..

[45] Grabowska K., Szczuko P. (2015). Ship resistance prediction with Artificial Neural Networks. Proceedings of the Signal Processing: Algorithms, Architectures, Arrangements, and Applications (SPA).

[46] Le D., Huang W., Johnson E. (2018). Neural network modeling of monthly salinity variations in oyster reef in Apalachicola Bay in response to freshwater inflow and winds. Neural Comput. Appl..

[47] Luenberger D.G. (1973). Introduction to Linear and Nonlinear Programming.

[48] Yildizel S.A., Arslan Y. (2018). Flexural strength estimation of basalt fiber reinforced fly-ash added gypsum based composites. J. Eng. Res. Appl. Sci..

[49] Argiz C., Menéndez E., Sanjuán M.A. (2013). Effect of mixes made of coal bottom ash and fly ash on the mechanical strength and porosity of Portland cement. Mater. Construcción.

[50] Sanjuán M.Á., Estévez E., Argiz C., del Barrio D. (2018). Effect of curing time on granulated blast-furnace slag cement mortars carbonation. Cem. Concr. Compos..

[51] Kumar Poloju K. (2017). Properties of Concrete as Influenced by Shape and Texture of Fine Aggregate.

[52] Chinchillas-Chinchillas M.J., Corral-Higuera R., Gómez-Soberón J.M., Arredondo-Rea S.P., Jorge L., Acuña-Aguero O.H., Rosas-Casarez C.A. (2014). Influence of the Shape of the Natural Aggregates, Recycled and Silica Fume on the Mechanical Properties of Pervious Concrete.

[53] Mehnert A., Jackway P. (1997). An improved seeded region growing algorithm. Pattern Recognit. Lett..

[54] Huang G.-B., Saratchandran P., Sundararajan N. (2004). An efficient sequential learning algorithm for growing and pruning RBF (GAP-RBF) networks. IEEE Trans. Syst. Man Cybern. Part B.

[55] Arora P., Varshney S. (2016). Analysis of k-means and k-medoids algorithm for big data. Procedia Comput. Sci..

[56] Dariane A.B., Azimi S. (2016). Forecasting streamflow by combination of a genetic input selection algorithm and wavelet transforms using ANFIS models. Hydrol. Sci. J..

[57] Tabakhi S., Moradi P., Akhlaghian F. (2014). An unsupervised feature selection algorithm based on ant colony optimization. Eng. Appl. Artif. Intell..

[58] Song Y., Liang J., Lu J., Zhao X. (2017). An efficient instance selection algorithm for k nearest neighbor regression. Neurocomputing.

[59] Yu H., Reiner P.D., Xie T., Bartczak T., Wilamowski B.M. (2014). An incremental design of radial basis function networks. IEEE Trans. Neural Netw. Learn. Syst..

[60] Iqbal S.Z., Gull H., Ahmed J. Incremental Sorting Algorithm. Proceedings of the Second International Conference on Computer and Electrical Engineering.

[61] Gurbuzbalaban M., Ozdaglar A., Parrilo P.A. (2017). On the convergence rate of incremental aggregated gradient algorithms. SIAM J. Optim..

